# Ultrafine fully vulcanized natural rubber modified by graft-copolymerization with styrene and acrylonitrile monomers

**DOI:** 10.1186/s40643-022-00577-5

**Published:** 2022-08-20

**Authors:** Krittaphorn Longsiri, Phattarin Mora, Watcharapong Peeksuntiye, Chanchira Jubsilp, Kasinee Hemvichian, Panagiotis Karagiannidis, Sarawut Rimdusit

**Affiliations:** 1 Research Unit in Polymeric Materials for Medical Practice Devices, Department of Chemical Engineering, Faculty of Engineering, Chulalongkorn University, Bangkok, 10330 Thailand; 2 Department of Chemical Engineering, Faculty of Engineering, Srinakharinwirot University, Nakhonnayok, 26120 Thailand; 3 Thailand Institute of Nuclear Technology, Nakhonnayok, 26120 Thailand; 4 School of Engineering, Faculty of Technology, University of Sunderland, Sunderland, SR6 0DD UK

**Keywords:** Graft copolymer, DPNR-g-(PS-co-PAN), UFPNR, Electron beam vulcanization, Spray drying

## Abstract

This research aims to modify ultrafine fully vulcanized powdered natural rubber (UFPNR) prepared by emulsion graft-copolymerization with styrene (St) and acrylonitrile (AN) monomers onto deproteinized natural rubber (DPNR). The effects of monomers content and St/AN weight ratio on grafting efficiency and thermal stability of the developed DPNR-g-(PS-co-PAN) were investigated. The results showed that grafting efficiency was enhanced up to 86% with monomers content 15 phr and weight ratio St:AN 80:20. The obtained DPNR-g-(PS-co-PAN) was radiated by an electron beam at various doses, followed by a spray drying process to produce UFPNR. The obtained modified UFPNR particles irradiated at dose up to 300 kGy were relatively spherical with a particle size of approximately 4.4 µm. Furthermore, the degradation temperature of 5wt% loss (*T*_d5_) of UFPNR was found in the range of 349–356 °C. The results revealed that the modified UFPNR is suitable as a toughening filler for a broader spectrum of polymers.

**Graphical Abstract:**

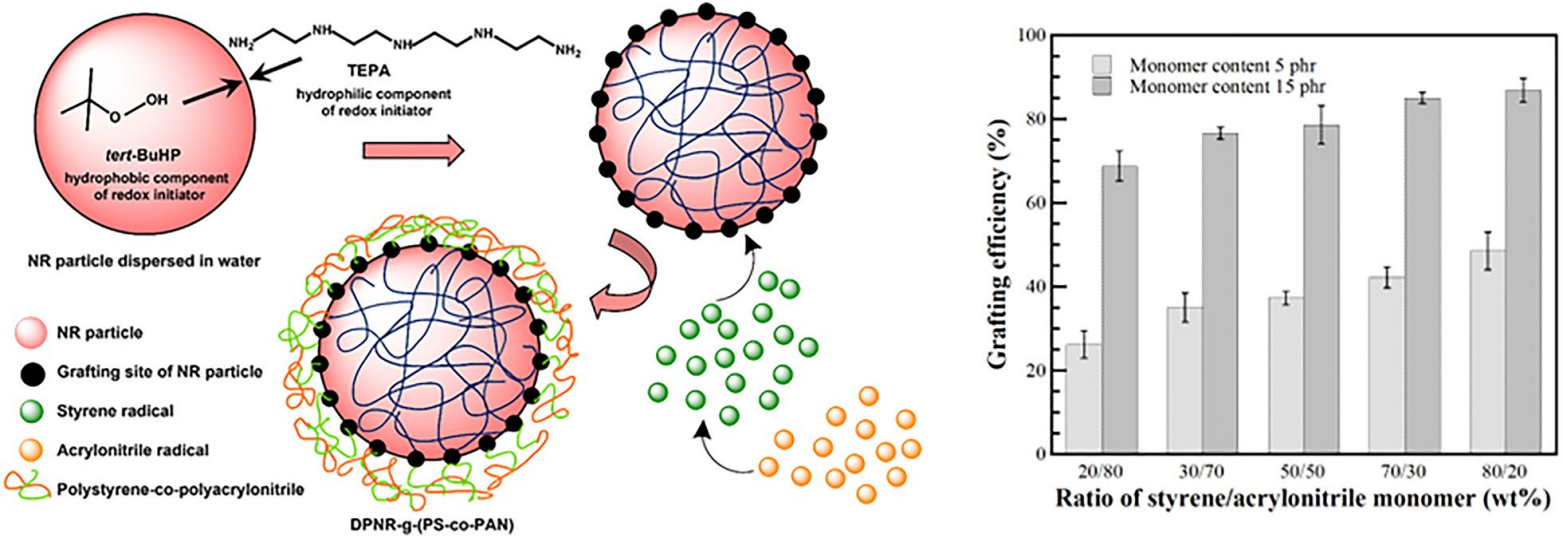

## Introduction

Recently, ultrafine fully vulcanized powdered natural rubbers (UFPNRs) have become an alternative to traditional commodity synthetic rubber powder, due to renewable resources used for their preparation, which are environmentally friendly and reasonably priced (Gupta et al. 2016; Haile et al. 2021; Yang et al. 2021). UFPNRs are illustrated to be suitable toughening fillers in a polymer matrix (Lin et al. 2021; Wongkumchai et al. 2021). Ultrafine fully vulcanized powdered rubbers (UFPRs) were prepared by irradiation vulcanization followed by a spray drying process to produce a controllable spherical particle powder (Qiao et al. 2002). Mostly UFPRs were obtained from synthetic rubber latexes as raw materials, such as St-butadiene rubber (SBR) (Liu et al. 2014), carboxylated-St butadiene rubber (XSBR) (Taewattana et al. 2018), AN–butadiene rubber (NBR) (Pan and Liu, 2022; Wu et al. 2010), and carboxylated-nitrile butadiene rubber (XNBR) (Huang et al. 2002; Taewattana et al. 2018). It is well-known that UFPRs show predominantly reinforcement effect into other rubbers (Tian et al. 2006) or other polymer matrices and modify properties of composites, such as reduction of the wear mass loss and friction coefficient of epoxy composite for friction materials (Yu et al. 2008), and increase of toughness or heat resistance of PVC (Wang et al. 2005), epoxy resin (Huang et al. 2002), polypropylene (Liu et al. 2004), and phenolic resin (Liu et al. 2006; Ma et al. 2005). UFPRs show good elasticity and are dispersed easily in polymer matrices during blending, as a result of their particular microstructure, i.e., a spherical powder form with high crosslinking on the particle surface and moderate crosslinking in the inner part (Tian et al. 2006). Moreover, the advantages of UFPRs over the conventional rubber in a latex form are higher stability upon long-term storage and not harmful to the human body during blending (Qiao 2020; Wang et al. 2019).

Despite the extensive use, unfortunately, the production of UFPNR is restricted because of aggregation between particles (Taewattana et al. 2018). Therefore, NR has been developed by enhancing the degree of crosslinking by adding polyfunctional monomers as a crosslinking agent or coagent in the rubber latex before the irradiation process. Lin et al. (2021) have studied the effect of acrylate coagents having different amounts of functional groups, i.e., dipropylene glycol diacrylate (DPGDA), trimethylol propane trimethaacrylate (TMPTMA), and ditrimethylol propane tetraacrylate (DTMPTA), on properties of UFPNR produced by radiation vulcanization and spray-drying. They suggested that DTMPTA, which has four functional acrylate groups, demonstrated high efficiency in enhancing the degree of crosslinking in NR that led to UFPNRs with much less agglomerated particles. However, NR which is a non-polar long chain hydrocarbon, lacks in some properties, i.e., it has poor solvent resistance, and limited application due to its immiscibility when blended with polar polymers (Arayapranee et al. 2002; Kangwansupamonkon et al. 2005). Therefore, it is necessary to modify the properties of NR before processing to overcome these problems.

Chemical modification by graft copolymerization is one of the most attractive techniques. The NR molecular structure, which contains cis-1,4-polyisoprene with an electron-donating methyl group attached to the carbon–carbon double bond in its main chain, can facilitate the reaction with other vinyl monomers and covalently bunched onto the NR backbone. Several vinyl monomers have been used for grafting modification of NR, such as St (Dung et al. 2016), AN (Prukkaewkanjana et al. 2013), methyl methacrylate (MMA) (Kongparakul et al. 2008), and maleic anhydride (MA) (Pongsathit and Pattamaprom 2018) to improve solvent resistance, thermal stability, mechanical properties, and compatibility of. Rimdusit et al. (2021) have improved thermal stability and solvent resistance of UFPNR via graft-copolymerization with St and or AN, respectively. The results revealed that the proper monomer content was 5 phr and proper radiation dose was 300 kGy for producing UFPNR-g-PS and UFPNR-g-PAN with maintaining rather high thermal stability. Therefore, the combination of St and AN monomer to form St/AN copolymer grafting on NR backbone is expected to improve the thermal stability and solvent resistance of NR and probably the compatibility with various polymer matrices having different polarity (Angnanon et al. 2011; Dung et al. 2017; Fukushima et al. 1988; Indah Sari et al. 2020; Nguyen Duy et al. 2020; Nguyen et al. 2019; Prasassarakich et al. 2001).

The present work is devoted on modifying UFPNR by graft-copolymerization with the combination of St and AN monomers onto deproteinized NR using tert-butyl hydroperoxide (TBHPO) and tetraethylenepentamine (TEPA) as a redox initiator. The effect of monomers content and St/AN weight ratios of DPNR-g-(PS-co-PAN) on monomers conversion and grafting efficiency were determined. The obtained DPNR-g-(PS-co-PAN) were irradiated by an electron beam in the presence of DTMPTA as coagent followed by spray drying process to produce UFPNR. The production of UFPNR-g-(PS-co-PAN) by grafting St-AN comonomers was studied to improve thermal stability and solvent resistance for using as toughening fillers. The effect of irradiation dose used on the morphology and thermal properties of UFPNR was also investigated.

## Experimental

### Chemicals

High ammonia natural rubber (HANR) latex containing 60% of dry rubber content (DRC) was obtained from Sri Trang Agro-Industry Public Co., Ltd. (Thailand). Sodium dodecyl sulfate (SDS; 99%, Merck), urea (99.5%), magnesium-sulfate heptahydrate (MgSO_4_·7H_2_O), St (99%), AN (99%), tert-butyl hydroperoxide (TBHPO; 70% in water), TEPA, DTMPTA, sodium hydroxide (NaOH), acetone (99.5%), and 2-butanone (99%) were purchased from Tokyo Chemical Industry Co., Ltd. (Tokyo, Japan).

### Preparation of DPNR latex and purification of St and AN monomers

DPNR was prepared by incubating HANR with 0.1 wt% urea and 1 wt% SDS at room temperature for 60 min, followed by centrifugation at 10,000 rpm, 15 °C for 30 min. After centrifugation, the cream fraction was redispersed in 0.5 wt% of SDS solution. The latex was washed repeated twice by centrifugation. The final product of DPNR latex was stabilized in 0.8 wt% of SDS solution and diluted to 30% DRC (Kawahara et al. 2004).

St and AN monomers were extracted with 10 wt% sodium hydroxide solution, washed with de-ionized water until neutral, and dried in MgSO_4_·7H_2_O to remove inhibitor (Dung et al. 2017).

### Graft copolymerization of DPNR with St and AN

The graft copolymerization of DPNR with St and AN in the latex state was conducted in 500 cm^3^ glass reactor, equipped with a mechanical stirrer, water bath and nitrogen gas inlet. The DPNR latex and SDS were charged into a glass reactor under a nitrogen atmosphere and stirring at the speed of 400 rpm, 40 °C for approximately 2 h. Afterwards, TEPA and TBHPO were used as an initiator at a concentration of 3.5 × 10^–5^ mol/g of dry rubber. St/AN monomers with a ratio 20/80, 30/70, 50/50,70/30 or 80/20 wt% was added in a solution of dry rubber at concentration 1.5 × 10^–3^ mol/g (15 phr) or 0.5 × 10^–3^ mol/g of dry rubber (5 phr). Then, the grafting reaction was continued for 2.5 h (Nguyen Duy et al. 2020).

After the reaction was finished, the unreacted monomers were removed by the rotary evaporator at 80 °C under reduced pressure for 1 h. The mixture was cast in a glass Petri dish and dried to constant weight in a vacuum oven at 50 °C. The monomers conversion of graft copolymerization reaction was determined by the gravimetric method, using the following equation: The increase in mass of grafted NR was equal to the amount of formed polymer and was used for the calculation of monomer conversion.

1 $${\text{Monomer}}{\text{s}}\text{ conversion }\,(\%) = \frac{\text{weight of polymer in gross copolymer}}{\text{total weight of monomers}} \times 100$$

After drying, the product was extracted with a mixture of acetone/2-butanone in ratio 3/1 v/v for 48 h using a Soxhlet apparatus to remove the free homopolymer, i.e., PS and PAN. The grafting efficiency was calculated as follows:

2 $$\text{Grafting efficiency }\,(\%) = \frac{\text{weight of polymer linked to NR}}{\text{total weight of the poly}\text{mer formed}} \times 100$$

### Preparation of ultrafine fully vulcanized powdered rubber (UFPNR)

The grafted DPNR was diluted to 20% DRC with de-ionized water in the presence of 3 phr of DTMPTA. The latex mixture was stirred for 15 min before being vulcanized by electron beam irradiation at the dose of 100, 200, or 300 kGy supported by Thailand Institute of Nuclear Technology (Public Organization). Then, the vulcanized grafted DPNR was dried by a spray dryer (model B-290 from BUCHI, Switzerland) with the inlet temperature at 150 °C, feed flow rate 7 mL/min, and air flow rate 500 L/hr. to achieve the ultrafine powdered rubbers as a bottom product.

### Samples characterization

The chemical structure of DPNR-g-(PS-co-PAN) obtained after Soxhlet extraction was studied using Fourier transform infrared spectroscopy (model 2000 FTIR, Perkin Elmer) with an attenuated total reflection (ATR) accessory (Waltham, Massachusetts, United States) in the range from 4000 to 600 cm^−1^, by averaging 128 scans at a resolution of 4 cm^−1^. ^1^H NMR spectra were recorded on a Bruker AV500D spectrometer 500 MHz (Bruker, Switzerland) and was used to confirm the FTIR results. The samples were dissolved in deuterated chloroform (CDCl_3_) using the pulse accumulation of 64 scans and LB parameter of 0.30 Hz.

The morphology of NR latex particles was observed with a transmission electron microscope (TEM, model JEM-1400 from JEOL Ltd., Tokyo Japan) with an accelerating voltage of 80 kV. Before observation, 1 mL of NR latex (30% DRC) was diluted using 300 mL deionized water and placed on a carbon-coated copper grid. The NR latex was stained with 1.0 wt% osmium tetroxide (OsO_4_) to enhance the resolution (Chueangchayaphan et al. 2017; Gosecka and Gosecki 2015; Schneider et al. 1996). After staining, the samples were dried in ambient air before observation.

After irradiation followed by spray drying process the obtained UFPNR was coated with thin gold using a JEOL ion sputtering device (model JFC-1200) for 4 min. The morphology was investigated by a scanning electron microscope (SEM, model JSM-6510A from JEOL Ltd., Tokyo Japan) with an accelerating voltage of 15 kV. The particle size of the UFPNR was measured using the Image J program.

The change of polarity of NR after chemical modification was estimated by their wettability. The rubber films were prepared by drying latex under reduced pressure at room temperature for a week and examined by static contact angle (sessile drop) measurements using a contact angle meter (model DM300, Kyowa Interface Science Co., Ltd., Japan). Distilled water was used as the test liquid. The shape of the drops was observed with a microscope equipped with a CCD camera, and the contact angles of the same sample were measured at least 5 times in ambient air, and an average value established.

The degradation temperature of the UFPNRs was evaluated using a thermogravimetric analyzer (model TGA1 module from Mettler-Toledo, Thailand). The samples ~ 10 mg was heated from 25 °C to 800 °C with a heating rate of 20 °C/min under nitrogen atmosphere at a nitrogen purge gas flow rate of 50 mL/min.

The glass transition temperature (*T*_g_) of UFPNR was determined using a differential scanning calorimeter (model DSC1 module from Mettler-Toledo, Thailand). The samples about ~ 10 mg was cooled to − 100 °C by liquid nitrogen and heated up to 25 °C with a constant rate of 10 °C/min under nitrogen atmosphere.

Swelling properties and gel content of UFRNR were then evaluated. The weight of UFPNR (*W*_1_) was measured before immersing in toluene (ρ_s_ = 0.87 g/cm^3^, *V*_1_ = 106.5 mL/mol) at room temperature for 24 h. After that, the swollen UFPNR was immediately weighted (*W*_2_), followed by drying in a vacuum oven at 80 °C for 24 h to remove the solvent and obtain the dried weight (*W*_3_). The swelling ratio (*Q*), molecular weight between crosslinks (*M*_c_), crosslink density (CLD) and gel fraction (*g*) were calculated using the following Eqs. (3–6) (Flory–Rehner equation) (Flory and Rehner 1943):

3 $$Q=\frac{(W2-W1)/{\uprho }_{s}}{W1/{\uprho }_{r}}.$$

4 $${M}_{C}=\frac{-{\uprho }_{r}{V}_{1}({{\mathrm{\varphi }}_{r}}^\frac{1}{3}-\frac{{\mathrm{\varphi }}_{r}}{2})}{Ln\left(1-{\mathrm{\varphi }}_{r}\right)+{\mathrm{\varphi }}_{r}+{X}_{12}{{\mathrm{\varphi }}_{r}}^{2}}; where {\mathrm{\varphi }}_{r}=\frac{1}{1+Q}.$$

5 $$\mathrm{CLD}=\frac{{\uprho }_{r}\mathrm{N}}{{M}_{C}}.$$

6 $$g=\frac{W3}{W1}$$

where *W*_1_, *W*_2_, and *W*_3_ are the weights of initial, swollen and dried samples, respectively. *ρ*_s_ and *ρ*_r_ are the densities of solvent (0.87 g/cm^3^ of toluene) and rubber, *ϕ*_r_ is the volume fraction of polymer in the swollen sample, *V*_1_ is the molar volume of the toluene solvent (106.5 mL/mol), χ_12_ is the polymer–solvent interaction parameter (the value of χ_12_ is 0.393 for toluene) and N is Avogadro's number (6.022 × 10^23^).

## Results and discussion

### Graft copolymerization of DPNR with St and AN

#### Effect of St/AN monomers ratio and monomers content on graft copolymerization

**Fig. 1:**
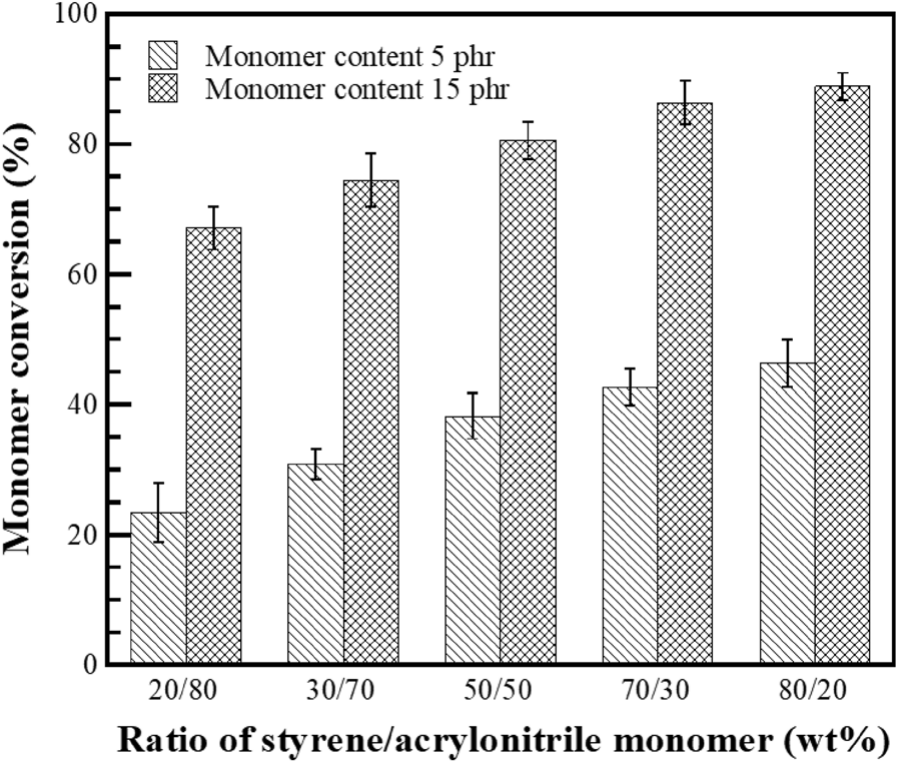
Variation of St/AN weight ratio as a function of monomer conversion (%) at different monomer content

**Fig. 2:**
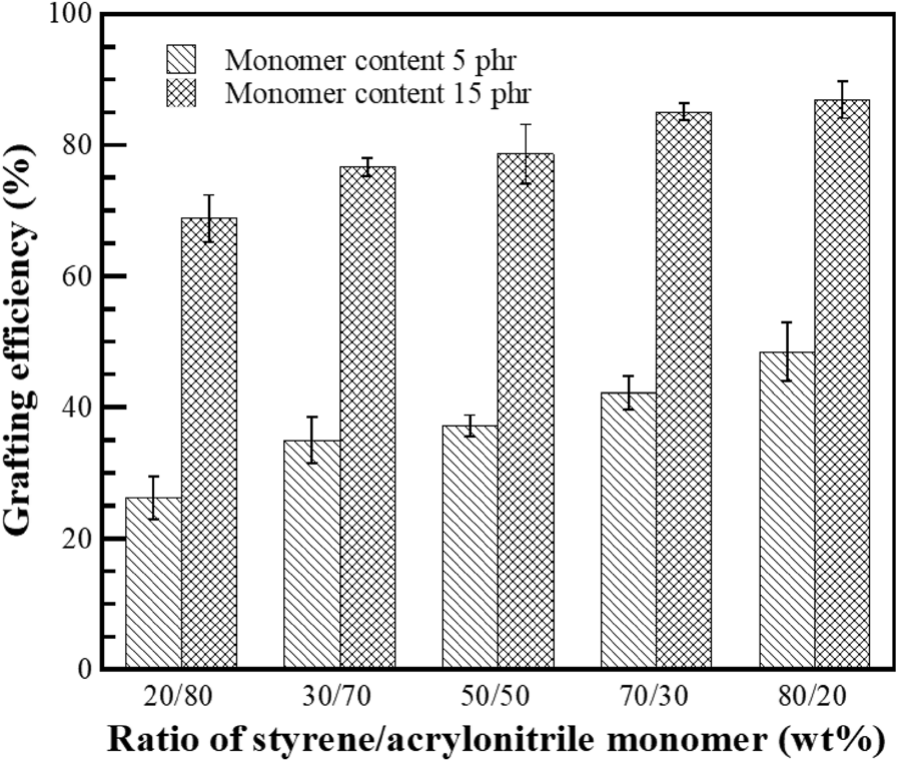
Variation of St/AN weight ratio as a function of grafting efficiency (%) at different monomers content

Monomers conversion of graft copolymerization reaction is the factor that evaluates the percentage of monomers converted to grafted copolymer and the amount of formed homopolymers. The grafting efficiency of process, expresses the PS-co-PAN actually attached to the poly-isoprene chains by chemical linkage. At the same time, the monomers can be homopolymerised under the reaction conditions; the reaction occurs in the aqueous phase and the homopolymers are formed as short-chain free polymer products, i.e., free-PS and free PN, eliminated by Soxhlet extraction with a suitable solvent. The monomers conversion and grafting efficiency of process in the presence of St/AN at various weight ratios were estimated by gravimetric analysis using Eqs. (1) and (2), respectively, as shown in Figs. 1 and 2. The monomer conversion at monomers content of 5 phr was found to be 23, 31, 38, 43, or 46%, whereas the grafting efficiency was 26, 35, 37, 42, or 48% with an addition of St/AN weight ratio of 20/80, 30/70, 50/50, 70/30, or 80/20, respectively. It was found that the monomers conversion and the grafting efficiency at monomer content of 5 phr was substantially increased with raising the amount of St. The most significant reason is that the solubility of the monomers in the aqueous and organic phase is responsible for the relative rate of monomer reaction. The St monomer being hydrophobic was similar to polarity of polyisoprene in NR. In contrast, AN monomer is a partially water-soluble monomer with relatively more affluent in the aqueous phase. For that reason, to increase the St monomer may preferentially react with polyisoprenyl macroradicals first to generate stable radical, which is styryl macroradicals (DPNR-g-St·) capable of further copolymerization with AN monomer (Arayapranee et al. 2002). Furthermore, St monomer can act as an electron donor to activate the carbon–carbon double bond of AN monomer, which is an electron acceptor by creating a charge transfer complex (CTC). In consequence, St-AN copolymers can be synthesized with the predominantly alternating structure to generate oligomer-radicals (AN-co-St·) which highly activates the weakly reactive double bond of AN towards the rubber macroradicals (Indah Sari et al. 2015; Ji et al. 2005). The monomers conversion and the grafting efficiency at monomers content 15 phr showed the same trend but higher than those at the content 5 phr. The monomers conversion at monomers content 15 phr increased from 67, 74, 80, 86, to 89, as well as the grafting efficiency was increased from 69, 77, 79, 85, to 86% with St/AN weight ratio 20/80, 30/70, 50/50, 70/30, or 80/20, respectively. It can be explained that during the copolymerization at higher monomers content more monomer and oligomer radicals are produced which raised the chance of the reaction between monomer and oligomer radicals with NR molecules to form graft copolymer.

#### Morphology study of DPNR-g-(PS-co-PAN) by TEM

**Fig. 3:**
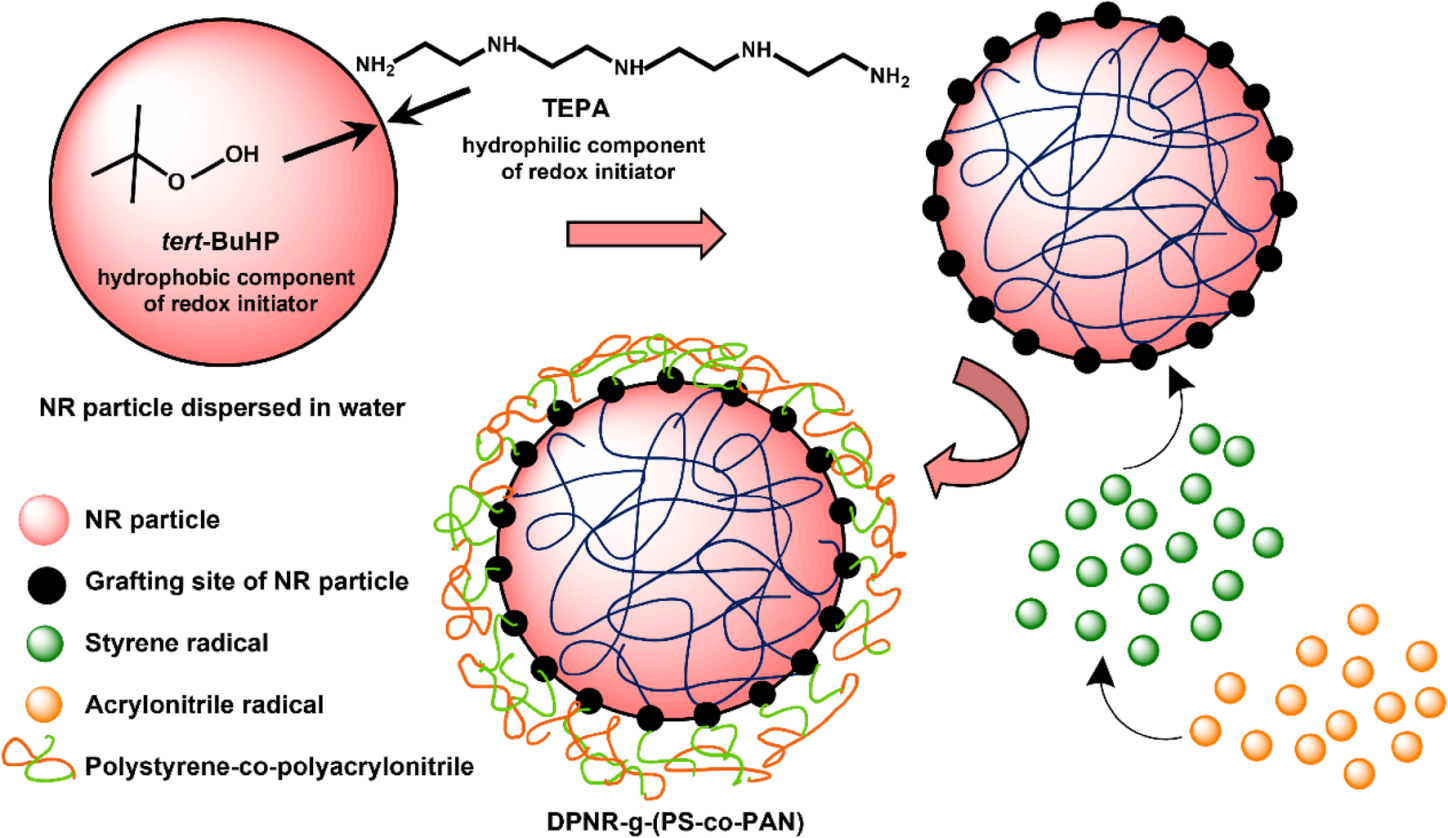
Possible reaction sites in the modified NR latex in the bipolar redox initiation systems (adapted from (Schneider et al. 1996))

**Fig. 4:**
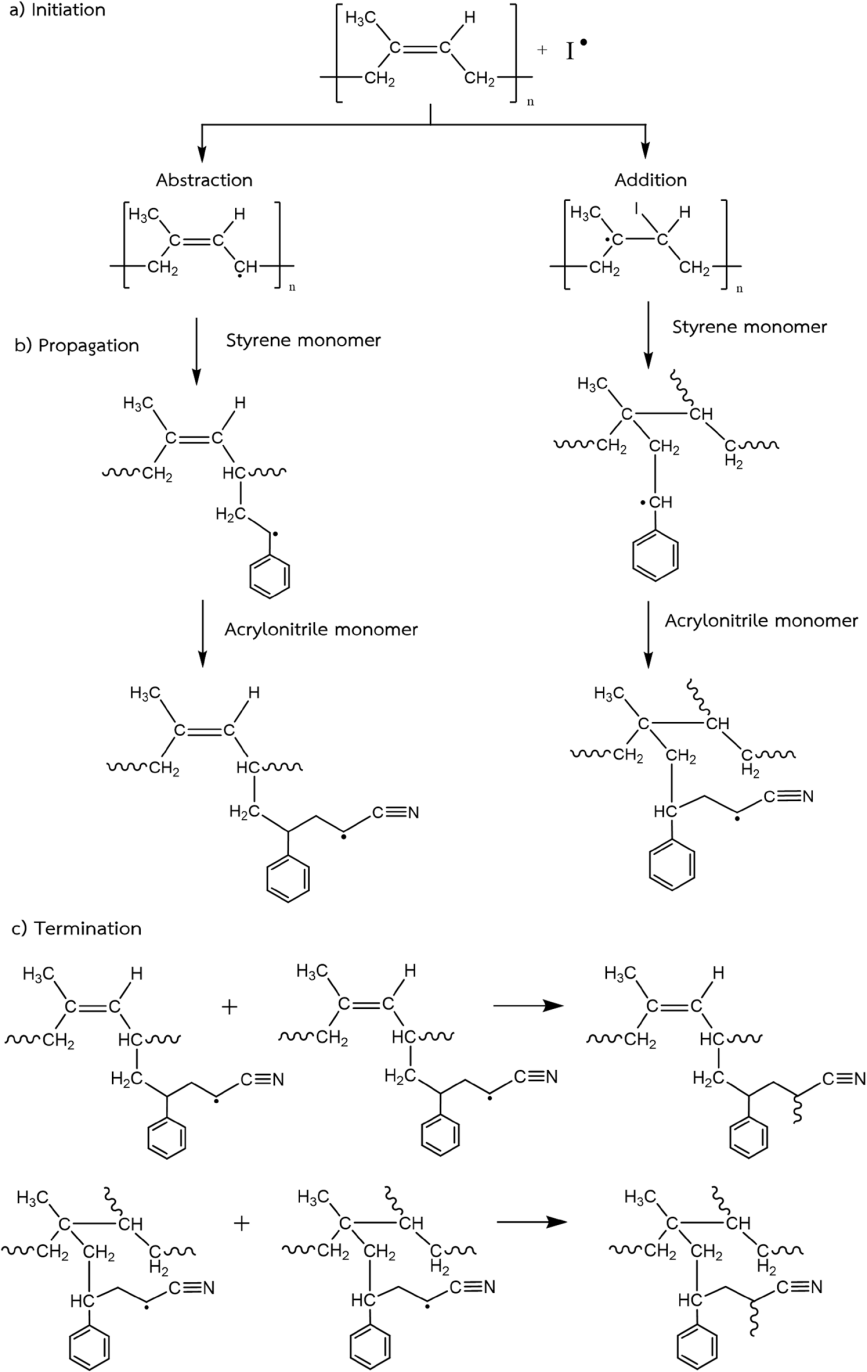
Mechanism of graft copolymerization studied

In the grafting process by emulsion copolymerization, the NR particles and droplets of undissolved monomers were stabilized by SDS surfactant molecules absorbed on their surfaces to form micelle particles. The emulsion copolymerization process takes place in the organic phase, which is inside the micelle particles, and very little occurs in the aqueous phase. The graft-copolymerization begins with the redox initiation system, which has two components, that is the TBHPO (a hydrophobic oxidizing agent) which prefers to remain strongly onto the NR surface and the TEPA (a hydrophilic reducing agent) which prefers to remain in the aqueous phase. These are proposed to be thermally dissociated to generate initiator radicals (I·) at the rubber/water interface and form grafting sites present on the surface of NR particles, as shown in Fig. 3 (Schneider et al. 1996). In Fig. 4 is shown the mechanism of graft copolymerization. In the initiation step (a) the initiator radicals possibly react with the active sites of the polyisoprene backbone in two ways. Through abstraction of an allylic hydrogen, which is the hydrogen in –CH_2_ next to carbon–carbon double bond and transfer of the radical to form an active site at allylic carbon that is a secondary polyisoprene macroradicals (DPNR). In addition, the initiator radicals might interact through an addition reaction with carbon–carbon double bond, breaking the double bond of rubber main chain to give a tertiary polyisoprene macroradicals (DPNR·) (Kochthongrasamee et al. 2006). At the same time, initiator radicals can attach to the St and AN monomers to form monomer and oligomer radicals, i.e., (St·), (AN·), and (AN-co-St·) similar to the proposed mechanism reported by (Staverman 1979). In the second step of propagation (b), a propagating polymer chain can be formed in the presence of monomer radicals and macroradicals to form graft copolymer. The cycle of the growing chain of the polymer particle continues until the monomer conversion is essentially complete in the termination step (c) by chain transfer to macromolecules or combination reaction (Azanam and Ong 2017).

**Fig. 5:**
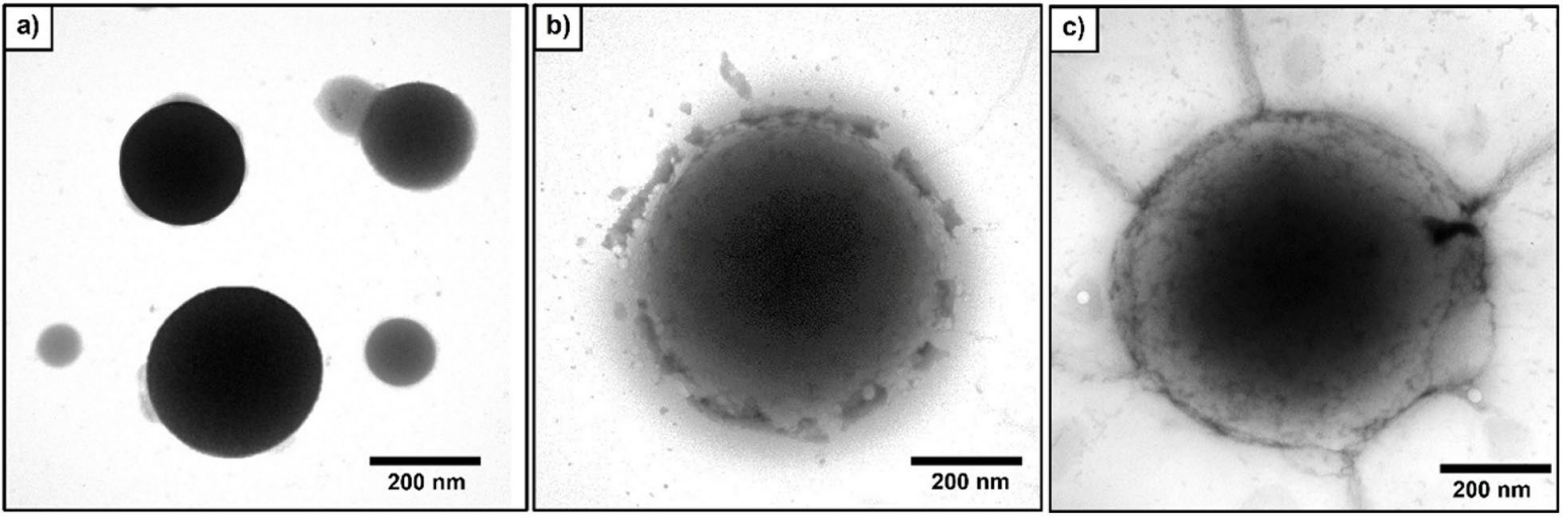
TEM micrographs of **a** Virgin NR **b** DPNR-g-(PS-co-PAN) at monomers content: 5 phr and **c** DPNR-g-(PS-co-PAN) at monomers content 15 phr (×100,000 magnification)

As mentioned previously, the characteristic morphology of grafted DPNR (DPNR-g (PS-co-PAN)) particles was confirmed by TEM micrographs compared to virgin NR. The micrograph of virgin NR particles is shown in Fig. 5a. The dark domains describe the electron-dens of the carbon–carbon double bonds of isoprene units inside the NR as spherical particles, with sharp edges and smooth surfaces (Chueangchayaphan et al. 2017; Gosecka and Gosecki 2015). Meanwhile, the micrographs of grafted DPNR particles with monomer contents at 5 and 15 phr are shown in Fig. 5b and c. The figures illustrated the contrast variation of electron density between the middle and the edge of particles. The dark domains at the middle represent the DPNR particles attributing to the core-structure, surrounded by the distinct brighter domains represent to the grafted PS-co-PAN phase attributing to the shell-structure (Schneider et al. 1996). The results suggest that the grafting copolymerization of St/AN was occurred on the DPNR surface and form the core–shell structure which is in a good agreement with the proposed mechanism of grafting-copolymerization. Moreover, the thickness of the shell layer is 1.1 ± 2.7 µm larger than that of virgin NR particles and forms a perfect shell at monomer content 15 phr ascribable to the highest grafting efficiency of grafted NR up to 86%. Since the active grafting sites are totally occupied at maximum grafting, a sufficient percent of grafting was required to keep the bonding between core and shell strong enough to prevent the breaking of core–shell particles at the interphase.

The advantages of core–shell structure may prevent the agglomeration of the DPNR particles led to processing aids when produced UFPNR and improved interfacial adhesion between UFPNR and polymer matrix.

#### Chemical structure study of DPNR-g-(PS-co-PAN)

**Fig. 6:**
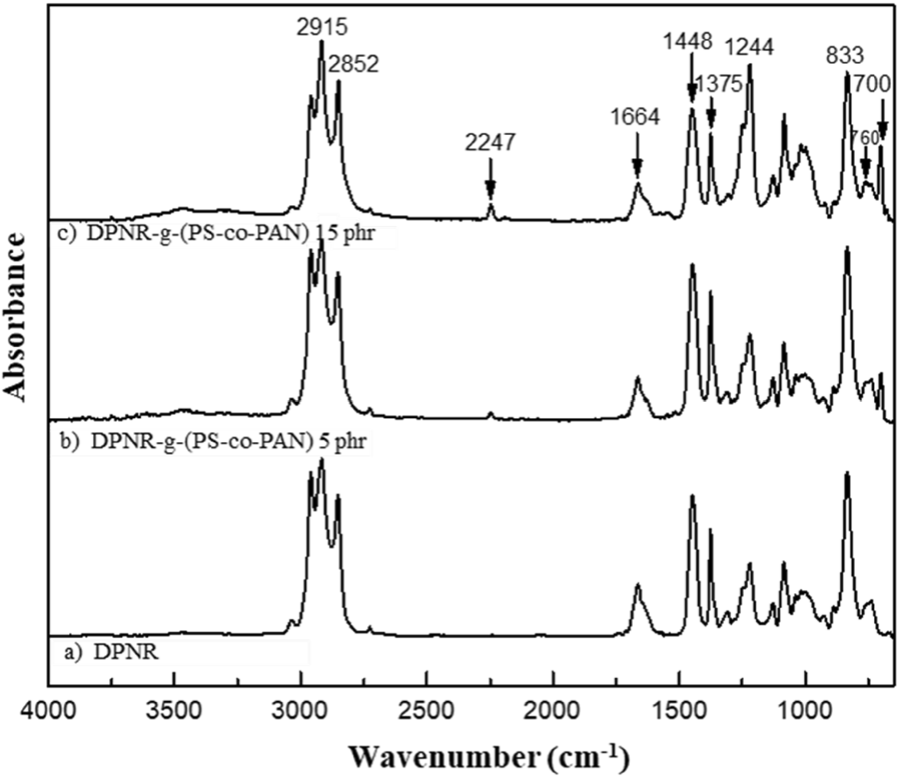
FTIR spectra of DPNR and DPNR-g-(PS-co-PAN) at different monomer content

The chemical structure of the grafted DPNR with St/AN monomers at weight ratio of 80/20, and with different monomers content 5 and 15 phr, which showed the highest monomers conversion and grafting efficiency was investigated by FTIR spectroscopy. The chemical structure of the obtained grafted DPNR after Soxhlet extraction was examined by comparing its FTIR signal with DPNR, and the results are plotted in Fig. 6. In all FTIR spectra the characteristic absorption bands of NR. In the spectrum of DPNR shown in Fig. 6a, the distinctive absorption peaks at 2915 and 2852 cm^−1^ corresponding to C–H stretching of –CH_3_ and –CH_2_–, respectively, and the absorption band at 1664 cm^−1^ corresponding to vibration of C=C stretching are shown. C–H stretching vibration at –CH_2_– is expected around 1448 cm^−1^ and the absorption band at 1375 cm^−1^ is assigned to C–H asymmetry vibration of –CH_3_. In addition, the absorption band at 1244 cm^−1^ is attributed to vibration of C–C stretching next to C=C, which is (R_2_C=CH–R) or at cis1,4 addition position, while the peak of 836 cm^−1^ indicated C=C bending vibration in NR main chain (Dinsmore and Smith 1948; Kishore and Pandey 1986; Nallasamy and Mohan 2004). Confirmation of grafted NR can be seen by considering the spectrum of DPNA-g-(PS-co-PAN) with monomers content 15 phr shown in Fig. 6c. It could be pointed out by the existence of characteristic absorption bands at 760 and 700 cm^−1^ which are related to vibration of C–H bending of styrenic benzene rings of polystyrene (Nguyen Duy et al. 2020). Furthermore, the new absorption band appeared at wavenumber 2247 cm^−1^ ascribed to vibration of C≡N stretching in PAN. In addition, the intensity of absorbance peak can imply the quantity of functional groups in graft copolymer. The intensity of absorption bands at 2247, 760, and 700 cm^−1^ obviously increased with increasing monomers content from 5 up to 15 phr. The intensity of the shoulder peak around 1650–1655 cm^−1^ and 833 cm^−1^ was decreased. This is due to the consumption of double bond (C=C) in NR structure during the grafting process.

**Fig. 7:**
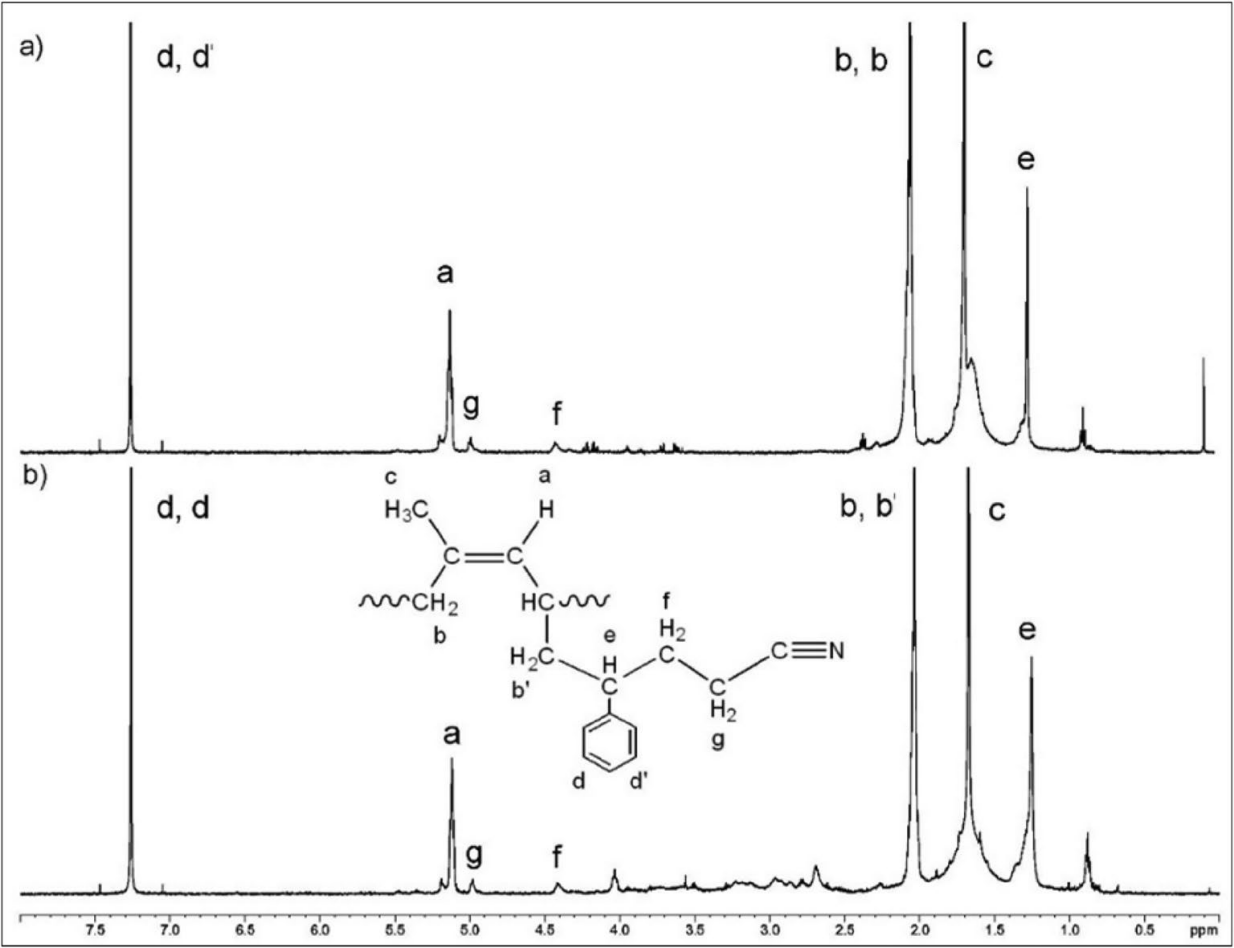
^1^H-NMR spectra of DPNR-g-(PS-co-PAN) at different monomer content

The chemical structure of DPNA-g-(PS-co-PAN) with monomer content 5 and 15 phr were also analyzed by ^1^H-NMR spectroscopy. The chemical shifts of NR at 1.61, 1.97 and 5.10 ppm, showed in Fig. 7 were attributed to the methyl proton CH_3_ (c), unsaturated CH_2_ (b, b’) and olefinic proton (a), respectively (Pongsathit and Pattamaprom 2018; Wongthong et al. 2013). Whereas the structure of St indicated the chemical shifts at 7.26 ppm attributed to the aromatic protons (d, d’) were found (Pukkate et al. 2007), and the chemical shifts at 1.3–1.4 ppm (e), which corresponds to the methylene protons (e) of DPNR linked to St in DPNR-g-(PS-co-PAN) was obtained (Liu et al. 2013). Moreover, the chemical shifts appeared at 4.45 and 4.90 ppm assigned to the backbone protons (f, g) of AN units (Prukkaewkanjana et al. 2013). These results confirmed that grafting NR with St and AN can form DPNR-g-(PS-co-PAN) structure.

Previous research (Rimdusit et al. 2021) showed that only graft copolymerization of NR with St or AN was not sufficiently crosslinked the NR molecules to reduce aggregated and tackiness between rubber particles and could not be produced modified UFPNR. Therefore, the suggestion is that only suitable high crosslinking density of NR particles by irradiation could produce UFPNR and solve the aggregation problem.

### Characterizations of ultrafine fully vulcanized NR grafted with polystyrene-co-polyacrylonitrile (UFPNR-*g*-(PS-*co*-PAN))

**Fig. 8:**
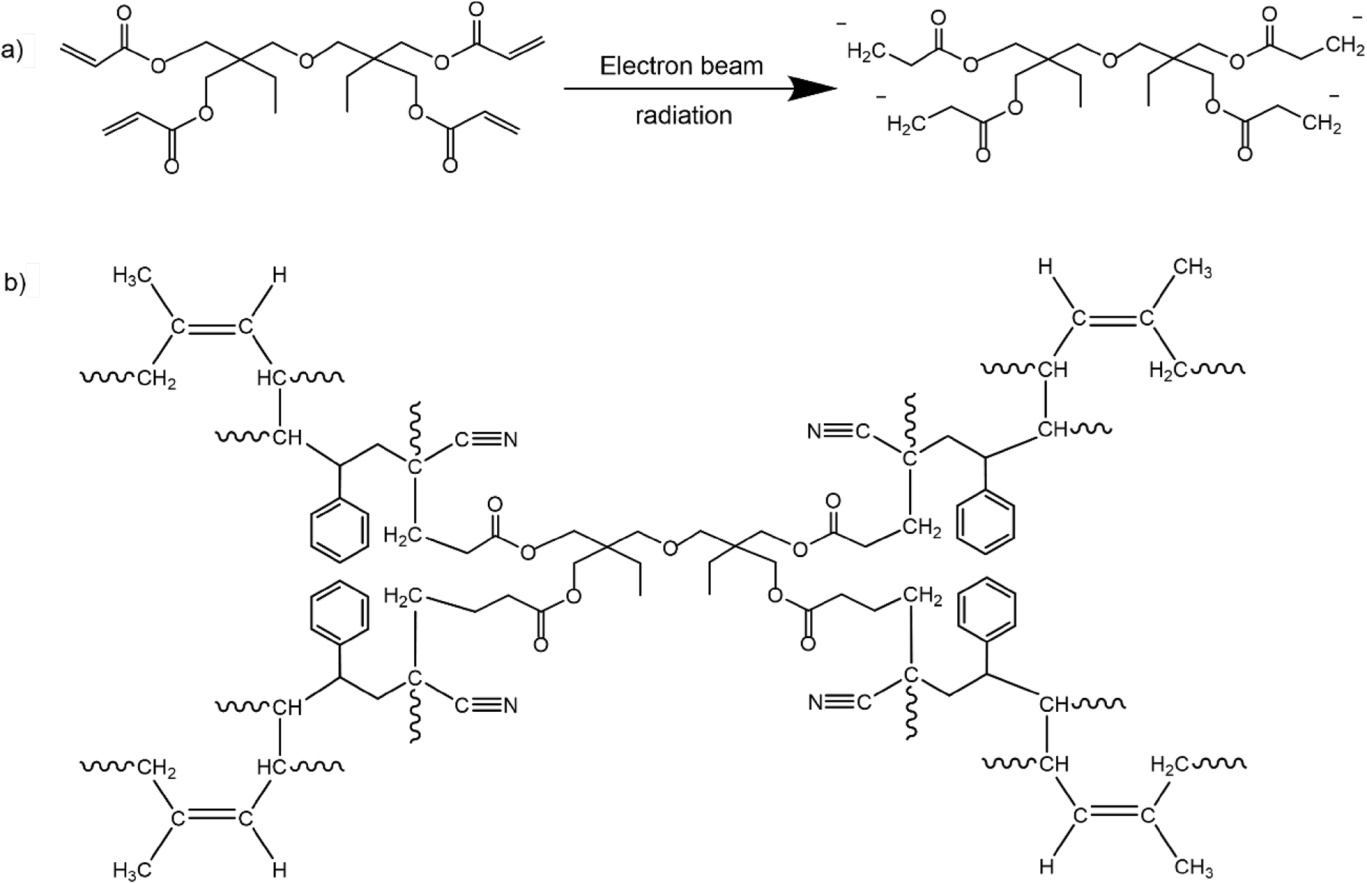
**a** DTMPTA structure was radiated by electron beam **b** possible structure of crosslinked UFPNR-g-(PS-co-PAN) in the presence of DTMPTA

The crosslinking process or vulcanization of NR molecules is obtained by electron beam vulcanization, which enhances crosslinking efficiency. Moreover, the crosslinking of NR can be further enhanced by adding a polyfunctional monomer, known as a crosslinking coagent. Previous research (Lin et al.) suggested that the addition of 3 phr of DTMPTA could enhance crosslinking density and produce the smallest particle size of UFPNR. Therefore, DTMPTA at 3 phr was used with electron beam irradiation in this research. The mechanism of DTMPTA irradiation by electron beam is shown in Fig. 8a. The electrons released from electron beam accelerator would attack π-electrons at the double bonds on tetra functional groups of DTMPTA to form monomer free radicals.

Meanwhile, electron beam irradiation also attacks π-electrons at the double bonds on NR structure to form polymeric free radicals. After that, the highly active free radicals can react to form chemical linkage between DTMPTA and the NR structure. There are two possible ways to promote the formation of a crosslinked network. First, the generated monomer free radicals would attach to the hydrogen at the AN segment on the grafted NR through hydrogen abstraction. Second, the generated monomer free radicals are attached with polymeric free radicals in NR chains to form crosslinked networks (Bee et al. 2018). The possible structure of crosslinked UFPNR-g-(PS-co-PAN) in the presence of DTMPTA to form three-dimensional crosslinking network illustrated in Fig. 8b.

#### The chemical structures of UFPNR-g-(PS-co-PAN)

**Fig. 9:**
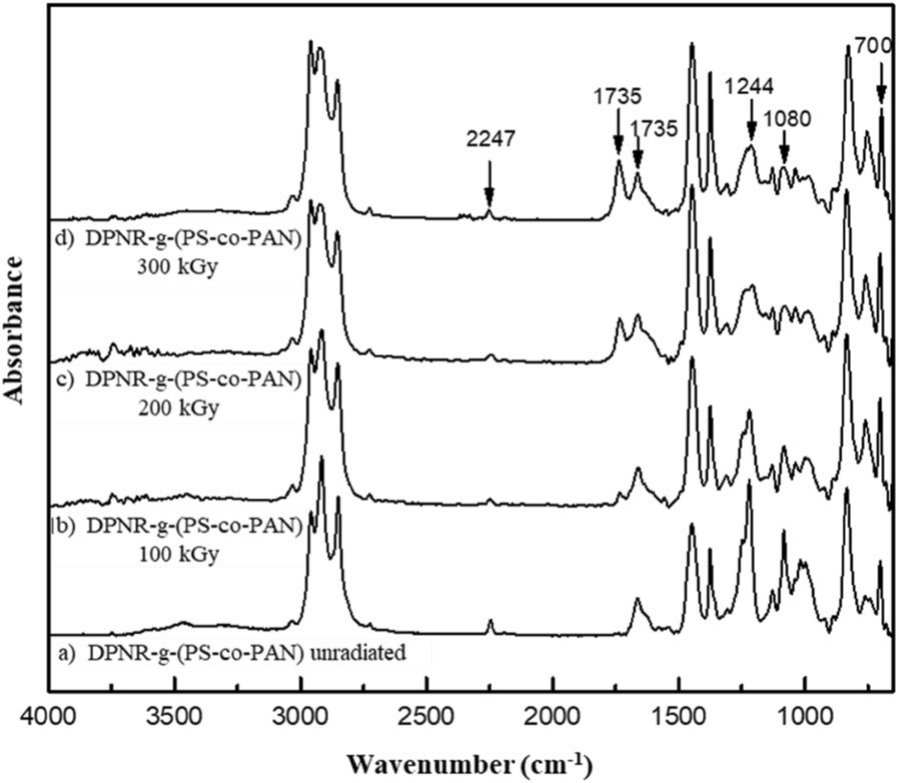
FTIR spectra of grafted NR at monomers content 15 phr with St/AN weight ratio 80/20 at various irradiation doses: **a** unradiated, **b** 100, **c** 200, and **d** 300 kGy

The obtained UFPNR-g-(PS-co-PAN) at monomers content 15 phr with St/AN weight ratio 80/20, which provided the highest grafting efficiency was irradiated at different irradiation doses, i.e., 100, 200, and 300 kGy. Their structure was studied by FTIR spectroscopy as illustrated in Fig. 9. The FTIR spectra showed the distinctive absorption peaks of NR at 836 cm^−1^ and 1650–1655 cm^−1^ corresponding to vibration of C=C bending and C=C stretching of NR main chain. In addition, the absorption bands at 1080 and 1244 cm^−1^ are attributed to vibration of C–C stretching next to C=C, which is (R_2_C=CH–R) or at cis1,4 addition position, while the peak at 836 cm^−1^ indicated C=C bending vibration in NR main chain. However, the intensity of the shoulder peak at 1650–1655 cm^−1^ corresponding to absorption peaks 1080 and 1244 cm^−1^ was decreased with increasing irradiation dose indicating the consumption of double bond (C=C) in NR structure to form C–C in three-dimensional crosslinking network. In addition, the absorbance peak in range of 1755–1745 cm^−1^ exhibited the presence of ester group of DTMPTA which promotes the crosslinking during irradiation vulcanization. In addition, the additional high energy of irradiation decreased the intensity of absorption peak at 2242 cm^−1^ referred to nitrile group C≡N of AN which was broken to form C=N bond (Badawy and Dessouki 2003). From the results of FT-IR spectroscopy study it can be concluded that NR grafted with St/AN copolymer can be vulcanized to form three-dimensional crosslinking network by addition of DTMPTA and concurrent electron beam irradiation.

#### The effect of electron beam irradiation on gel formation and swelling behaviors of UFPNR-g-(PS-co-PAN)

**Fig. 10:**
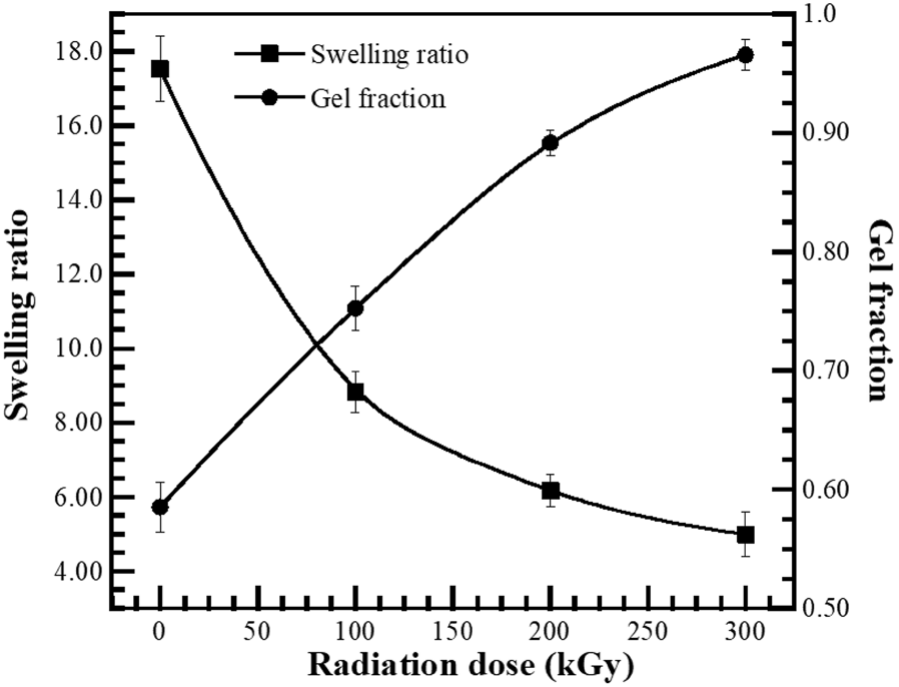
(Black square) Swelling ratio and (Black circle) gel fraction of UFPNR-g-(PS-co-PAN) 15 phr with ST/AN weight ratio 80/20 at various irradiation doses

The swelling behavior of NR was evaluated in toluene to determine the ability to resist against this when immersed for 24 h at room temperature. The parameters of swelling behaviour which determine the degree of crosslinking are the swelling ratio (Q), molecular weight between crosslinks (M_c_), crosslinking density (CLD) and gel fraction (g) based on Eqs. 3–6, respectively. The effect of used irradiation dose (100, 200, and 300 kGy) on these parameters for grafted NR at monomers content of 15 phr with St/AN 80/20 weight ratio are plotted in Fig. 10, and the numerical data are tabulated in Table 1. The results shown a significantly decreased of swelling ratio from 17.52 ± 0.88 to 8.83 ± 0.54 when grafted NR was radiated with an electron beam 100 kGy. Moreover, the swelling ratio of the grafted NR was decreased from 6.57 ± 0.44 to 4.99 ± 0.59 when the irradiation dose increased from 200 to 300 kGy, respectively. This can be explained by the sufficient electron beam irradiation which can activate the π-electrons of the double bonds and form highly active free radicals and a three-dimensional crosslinked network. Therefore, toluene molecules are more difficult to penetrate into the NR molecules. While the gel fraction continuously increased with the increase of irradiation dose. The results showed that the gel fraction increased from 0.58 ± 0.02 to 0.75 ± 0.02 and continues to increase from 0.89 ± 0.01 to 0.96 ± 0.01 when the irradiation dose increased from 200 to 300 kGy, respectively. This phenomenon was attributed to the long straight chain of NR, which has solubility parameter nearly that of toluene, while the molecules of the grafted NR chain have strong interaction with the toluene molecules which cause the expanding of NR chains and the swelling in the solvent eventually. Therefore, the increment of irradiation doses is extremely influential to gel fraction cause; it leads to increased inter-molecular crosslink between NR chains to form a three-dimensional crosslinking network that resists solvent penetration (Manshaie et al. 2011; Tuti et al. 2015).

**Table 1:** Swelling ratio, gel fraction, molecular weight between crosslinks and crosslink density of UFPNR-g-(PS-co-PAN)

UFPNR-g-(PS-co-PAN)	Swelling ratio (*Q*)	Gel fraction (*g*)	*M*_c_ (g/mol)	CLD (mol/cm^3^)
Unirradiation	17.52 ± 0.88	0.58 ± 0.02	93,598 ± 8,388	0.83 × 10^–22^ ± 0.09
100 kGy	8.83 ± 0.54	0.75 ± 0.02	27,336 ± 7,022	1.54 × 10^–22^ ± 0.09
200 kGy	6.57 ± 0.44	0.89 ± 0.01	14,161 ± 4,610	2.13 × 10^–22^ ± 0.07
300 kGy	4.99 ± 0.59	0.96 ± 0.01	10,434 ± 2,932	2.45 × 10^–22^ ± 0.05

#### The effect of electron beam irradiation on molecular weight between crosslinks and crosslink density of UFPNR-g-(PS-co-PAN)

**Fig. 11:**
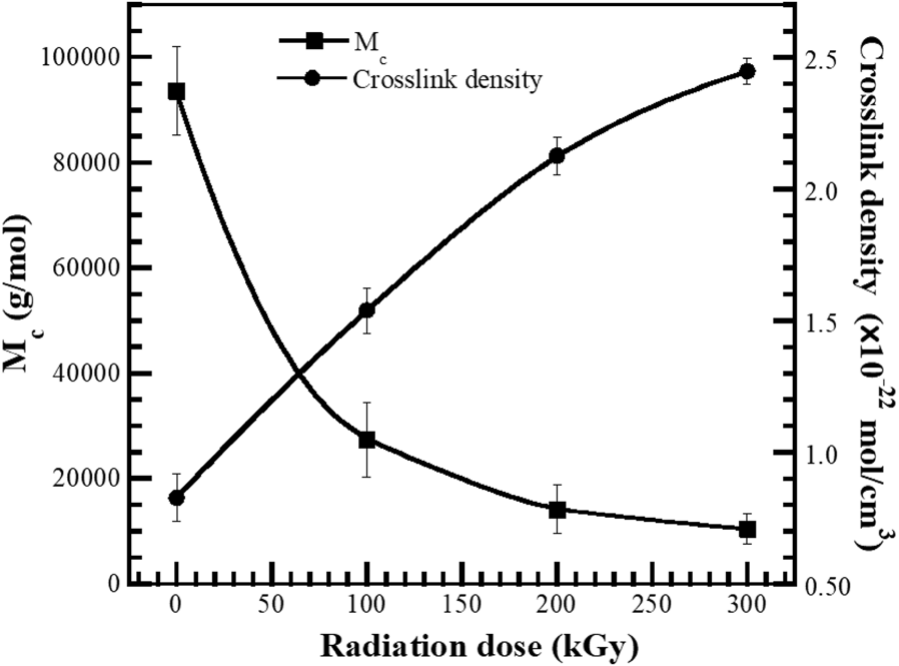
(Black square) Molecular weight between crosslinks (*M*_c_) and (black circle) crosslink density of UFPNR-g-(PS-co-PAN) 15 phr with St/AN weight ratio 80/20 at various irradiation doses

The effect of irradiation dose on the crosslink density, and molecular weight between crosslinks of grafted NR at monomer content 15 phr with St/AN 80/20 weight ratio after irradiation at 100, 200, and 300 kGy, are plotted in Fig. 11. The crosslink density of unirradiated grafted NR was 0.83 × 10^–22^ ± 0.09, whereas crosslink density values of radiated grafted NR increased from 1.54 × 10^–22^ ± 0.09, 2.13 × 10^–22^ ± 0.07 and 2.45 × 10^–22^ ± 0.05 with increasing irradiation dose from 100, 200, or 300 kGy, respectively. Due to the influence of higher energy irradiation doses, the thermal energy stimulated rubber latex to promote more free radicals, which are more suitable to form a three-dimensional crosslinking network. This behavior causes hindrance of solvent penetration into NR molecules leading to reduce the swelling ratio. While, as expected, the molecular weight between crosslinks decreased with increasing the irradiation dose.

#### The effect of irradiation dose on morphology of UFPNR-g-(PS-co-PAN)

**Fig. 12:**
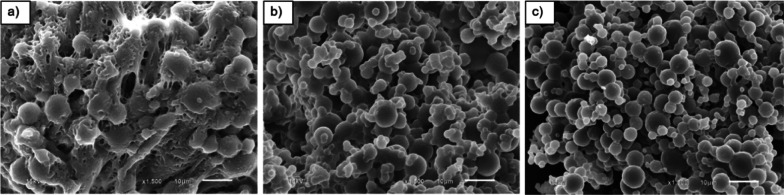
SEM micrographs (×1500 magnification) of UFPNR-g-(PS-co-PAN) at monomers content 5 phr with various irradiation doses: **a** 100 kGy, **b** 200 kGy, **c** 300 kGy

In this section, the effects of irradiation doses on morphology of UFPNR-g-(PS-co-PAN) with St/AN weight ratio 80/20 were investigated due to the highest grafting efficiency at monomer contents of 5 and 15 phr which could result in thermal stability and solvent resistance. The grafted NR with St/AN weight ratio 80/20 having the highest grafting efficiency at monomer contents of 5 and 15 phr was prepared by irradiation with electron beam of 100, 200, or 300 kGy, followed by spray drying process to produce modified UFPNRs. The morphology of modified UFPNRs was observed by SEM. SEM micrographs of the modified UFPNR with monomer content of 5 phr with irradiation dose at 100 kGy are shown in Fig. 12a. The modified UFPNR still shows aggregated and tacky rough surfaces and uncertain spherical particles, because of the amount of electron beam, which was not sufficient enough to generate free radicals for crosslinking. When increasing the irradiation dose to 200 and 300 kGy as shown in Fig. 12b, c, respectively, the aggregation of modified UFPNR tended to be less at higher irradiation doses, and the particles showed the least aggregation at irradiation dose of 300 kGy.

**Fig. 13:**
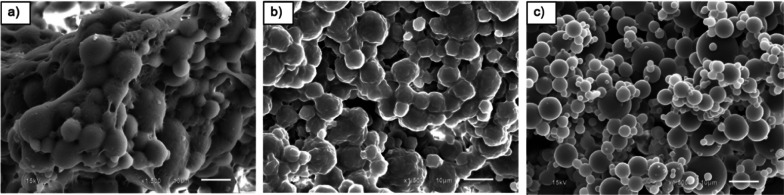
SEM micrographs (×1500 magnification) of UFPNR-g-(PS-co-PAN) at monomers content 15 phr with various irradiation doses: **a** 100 kGy, **b** 200 kGy, **c** 300 kGy

Figure 13a shows SEM micrographs of the modified UFPNR with monomer content 15 phr at irradiation dose 100 kGy. The particles of UFPNR have smoother surface when compared to modified UFPNR with monomer content of 5 phr with the same irradiation dose (Fig. 12a). It is worth noting that the monomers conversion and the grafting efficiency at monomer content 15 phr are higher than those at 5 phr. Furthermore, the results confirmed that copolymers of St and AN at monomer content 15 phr were sufficiently grafted on to the rubber surface reducing aggregation and tackiness between rubber particles permitting the production of modified UFPNR. However, particles still showed aggregation, high tackiness and uncertain spherical. When increasing the irradiation dose at 200 and 300 kGy, as shown in Fig. 13b, c, respectively, the aggregation of modified UFPNR tended to be less at higher irradiation doses and the particles showed the least aggregation at irradiation dose of 300 kGy. These results show the influence of irradiation dose on free radicals generation, which form a more dense three-dimensional crosslinking network. This results to reduced tackiness between of rubber particles surface reduced aggregation and more smooth surface particles, along with the smaller particle sizes (Rezaei Abadchi and Jalali-Arani 2014). The increase of doses resulted in a systematically decrease in the resulting rubber particle size. This phenomenon is attributed to the simultaneous mainchain scission and crosslinking of the rubber macromolecules occur during irradiation process particularly at higher dose of electron beam. From SEM micrographs, and the swelling behaviour study, it can be concluded that the increment of electron beam irradiation at 300 kGy is suitable for the production of modified UFPNR. Moreover, the particle sizes of the developed UFPNR-g-(PS-co-PAN) are smaller than that of UFPNR-g-PS or UFPNR-g-PAN investigated by Rimdusit et al. with the average sizes of 5.95 ± 3.03 µm and 6.39 ± 2.71 µm, respectively (Rimdusit et al. 2021).

#### The effect of grafting efficiency on morphology of UFPNR-g-(PS-co-PAN)

**Fig. 14:**
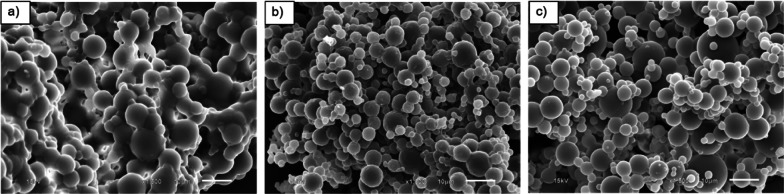
SEM micrographs (×1500 magnification) of **a** virgin UFPNR and UFPNR-g-(PS-co-PAN) at monomer content **b** 5 and **c** 15 phr at irradiation dose 300 kGy

The virgin NR and the grafted NR with St/AN weight ratio 80/20 with monomers contents at 5 and 15 phr were irradiated with electron beam of 300 kGy absorption dose, followed by spray drying process to produced UFPNR and modified UFPNRs. Figure 14 shows their SEM micrographs; aggregation of rubber particles was observed in virgin UFPNR which tended to fuse with each other, although at increment irradiation dose up to 300 kGy. Different morphology in modified UFPNR at monomers content 5 and 15 phr is observed in Fig. 14b, c, respectively. It can be seen that the morphology of modified UFPNR show non-aggregated and relatively spherical particles with relatively smooth surface and similar uniform particle size distribution. The surface of the UFPNR-g-(PS-co-PAN) at monomers content of 15 phr was smoother than that of the UFPNR-g-(PS-co-PAN) at monomers content of 5 phr. It is probable due to formation of a denser crosslinked network and the modification of the rubber surface, reducing the tackiness of rubber particles and resulting in less aggregated and smoother surface particles (Rimdusit et al. 2021).

**Fig. 15:**
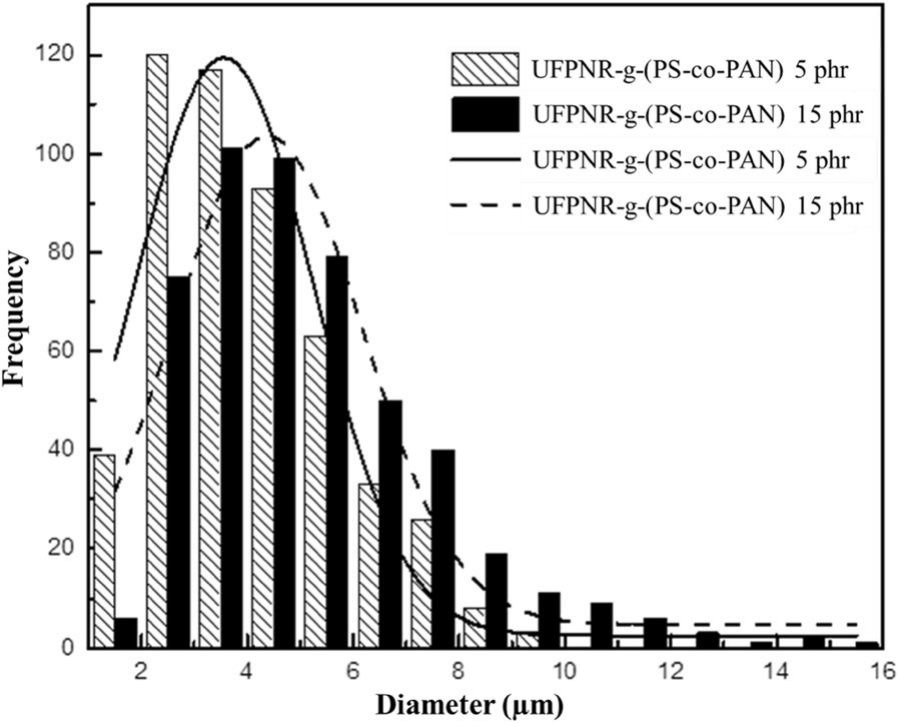
Particle sizes of the modified UFPNR with monomer content (square with upper left to lower right fill) 5 and (black square) 15 phr at irradiation doses 300 kGy

The average particle sizes of the modified UFPNRs were measured from 500 particles, the results obtained are shown in Fig. 15, and the numerical data are tabulated in Table 2. It was found that the average particle sizes of the modified UFPNR were 3.56 ± 1.70 and 4.38 ± 1.79 µm at monomer content 5 or 15 phr, respectively; due to higher grafting efficiency at monomer content, 86% for 15 phr than 48% for 5 phr. This may be the reason that the particles are slightly larger.

**Table 2:** Particle size of the modified UFPNR

Monomer contents (phr)	St/AN monomer (wt%)	Grafting efficiency (%)	Irradiation doses (kGy)	Average particle size (µm)
5	80/20	48	300	3.56 ± 1.70
15	80/20	86	300	4.38 ± 1.79

#### The effect of monomer ratio on surface properties of UFPNR-g-(PS-co-PAN)

**Fig. 16:**
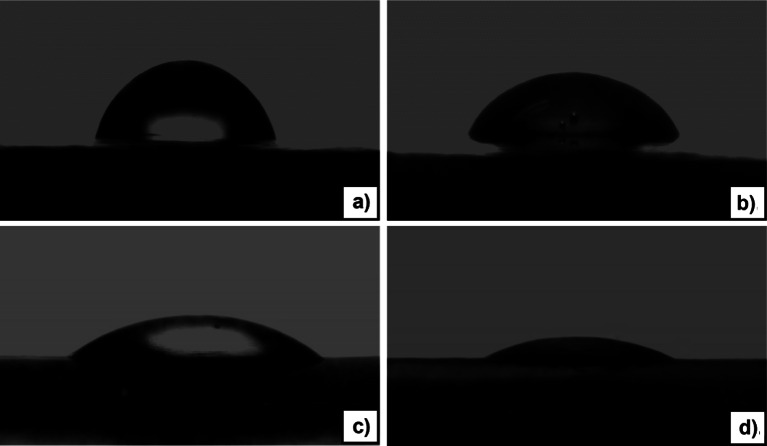
Contact angles of a water droplet on surface of NR films after irradiation **a** unmodified NR and grafted NR at monomer content 15 phr with various St/AN monomers weight ratios; **b** 80/20, **c** 50/50, and **d** 20/80 wt%

The measurement of water contact angle is one of the conventional methods used for estimating the hydrophilicity of polymer surfaces. PS-co-PAN was purposely grafted to the NR molecule to improve the affinity of NR with polar surfaces. Profiles of water contact angle on the surfaces of unmodified NR and grafted NR are illustrated in Fig. 16. The contact angle of NR showed the highest value 71 ± 0.1° indicating the lowest hydrophilicity (Cabrera et al. 2017). While compared to grafted NR, the contact angle of the films was reduced from 56 ± 0.1° to 40 ± 0.3° and the lowest contact angle appeared at 27 ± 0.7° by increasing the AN content from 20, 50, and 80 wt%, respectively. The decrease in the water contact angle of NR after grafting modification is attributed to the presence of grafted poly(St-co-AN) chains on to NR surface as mentioned before at TEM analysis. As the AN groups present in the grafted poly(St-co-AN) are capable of hydrogen bonding with water molecules, they facilitate the spreading and wetting of a water drop on an NR surface. This would lead to a noticeable reduction in the water contact angle, as the AN content increases, enhancing the hygroscopic characteristic of NR (Safeeda Nv et al. 2016).

#### The effect of grafting efficiency on thermal stability of UFPNR-g-(PS-co-PAN)

**Fig. 17:**
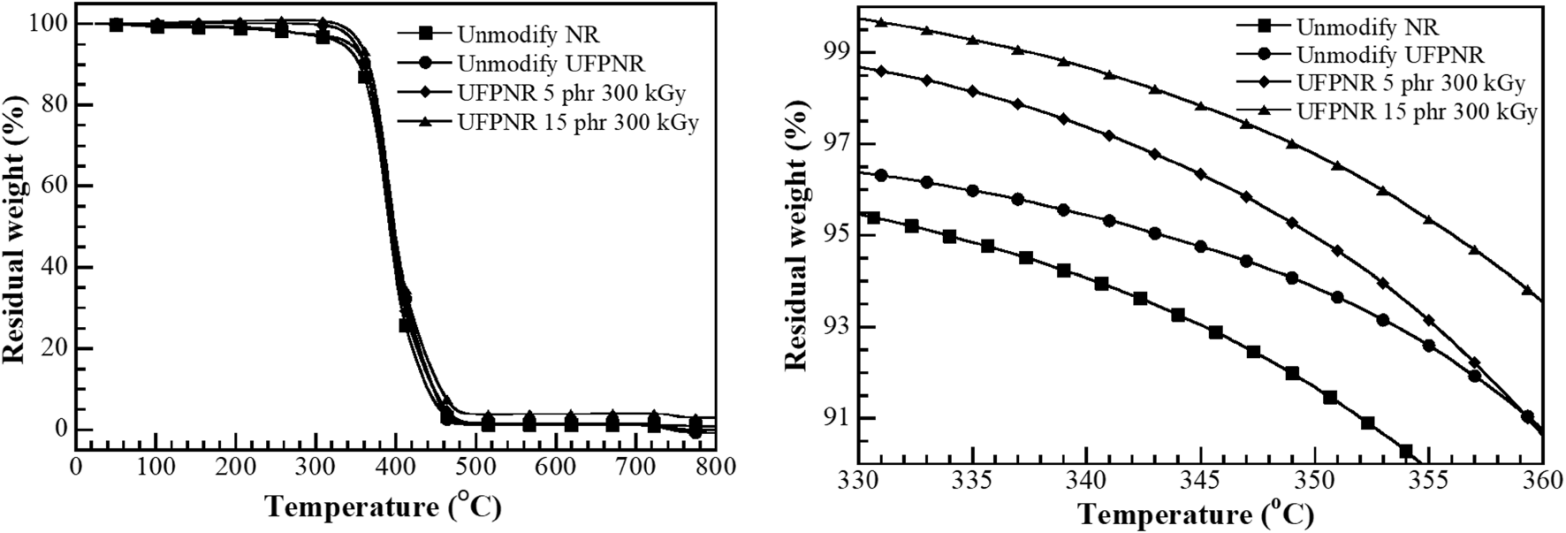
Degradation temperature at 5% weight loss (*T*_d5_) (black square) unmodified NR, (black circle) unmodified UFPNR at irradiation dose 300 kGy, and UFPNR-g-(PS-co-PAN) at monomers content (black diamond) 5 phr and (black up-pointing triangle) 15 phr at irradiation dose 300 kGy

The effect of grafting efficiency and irradiation dose on thermal stability, i.e., the degradation temperature at 5% weight loss (*T*_d5_) which is a vital property indicating their performance at elevated temperatures was studied. In Fig. 17, TGA thermograms of virgin UFPNR and the modified UFPNR are shown, and the numerical data are presented in Table 3. It was found that *T*_d5_ of the virgin NR without irradiation was 334 °C and increased to 344 °C after irradiation with 300 kGy. This is because the electron beam provides radical reactions by activating the double bond of NR structure and tetra-acrylate groups of DTMPTA to generate highly reactive radicals which form intermolecular and intramolecular C–C bonds in NR chains and a three-dimension network structure (Akiba and Hashim 1997; Dawes et al. 2007). Whereas C–C single bond is more stable than C=C double bonds, the crosslinks of NR chains can stabilize and restrict molecular mobility; more thermal energy is required to cause destructive changes leading to enhancement of *T*_d5_ (Bandzierz et al. 2018).

**Table 3:** Degradation temperature at 5% weight loss (*T*_d5_) at various monomer content

Sample	Monomer contents (phr)	ST/AN (wt%)	Irradiation doses (kGy)	*T*_d5_ (°C)
Unmodified NR	–	–	Unradiated	334
Unmodified UFPNR	–	–	300	344
UFPNR-g-(PS-co-PAN)	5	50/50	300	350
UFPNR-g-(PS-co-PAN)	15	50/50	300	356

Then, the modified UFPNRs' degradation temperature was evaluated by considering, *T*_d5_ of unmodified UFPNR and modified UFPNR under the same irradiation dose at 300 kGy. It was found that *T*_d5_ was increased from 344 of unmodified UFPNR to 350 and 356 °C for UFPNR-g-(PS-co-PAN) with monomer content 5 and 15 phr, respectively. It is worth to note that the thermal stability of the prepared UFPNR-g-(PS-co-PAN) is higher than that of the UFPNR-g-PS (343 °C) reported by (Rimdusit et al. 2021). Grafting of St-AN copolymer onto NR molecules improves *T*_d5_ of UFPNR besides the improvement of surface polarity of NR. Moreover, the free radicals generated during the irradiation process would abstract the hydrogen attached to the AN section in rubber structure to form a crosslinked network of PAN (Badawy et al. 2003; Park et al. 2014; Xue et al. 1997).

Furthermore, higher grafting efficiency at monomers content, 15 phr (i.e., 86%) observed than that of 5 phr (i.e., 48%). The increased amount of St, grafted on the NR backbone with the bulky side chains of aromatic rings resulted in greater difficulty for the polymer chains to flow or to slide past each other and generally led to a simultaneous increase of the chain stiffness of the modified UFPNRs. Therefore, the thermal stability was enhanced by grafting with PS-co-PAN (Seleem et al. 2017).

The obtained results showed that the modified UFPNRs by grafting with St/AN at monomers content 15 phr improved *T*_d5_ by 12 °C when compared to unmodified UFPNR, and improved *T*_d5_ by 22 °C when compared to virgin NR.

#### The effect of monomer ratio on thermal stability of UFPNR-g-(PS-co-PAN)

**Fig. 18:**
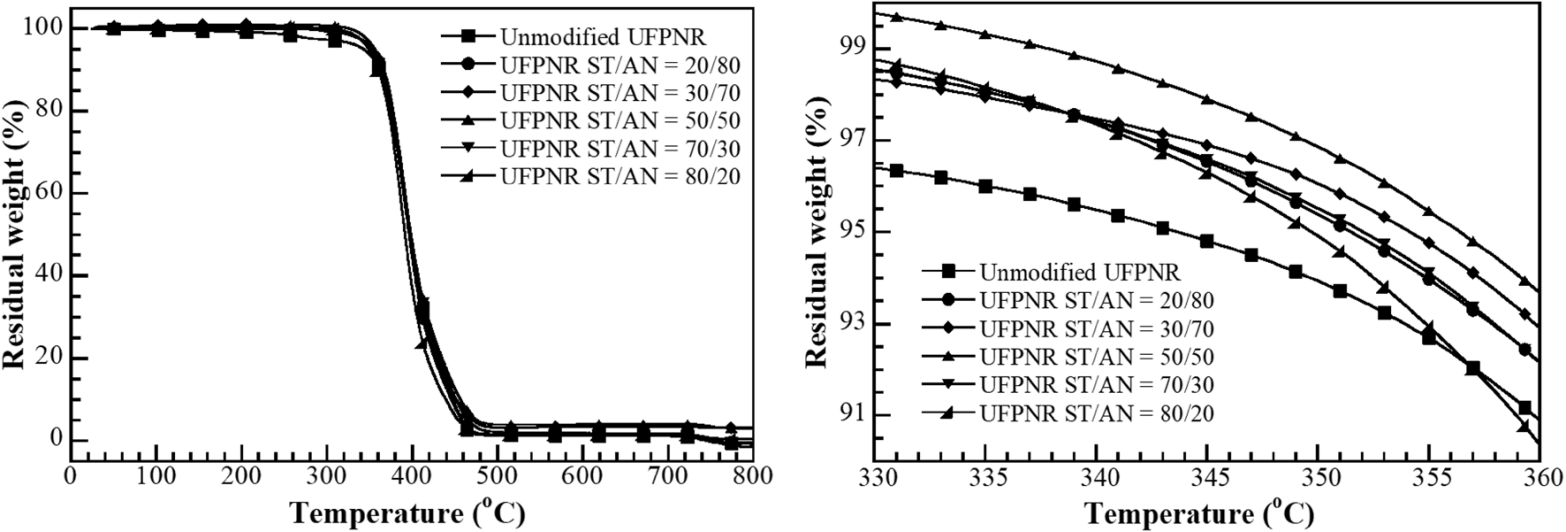
Degradation temperature at 5% weight loss (*T*_d5_) (black square) unmodified UFPNR at irradiation dose 300 kGy and UFPNR-g-(PS-co-PAN) at monomers content 15 phr with St/AN weight ratios: (black circle) 20/80, (black diamond) 30/70, (black up-pointing triangle) 50/50, (black down-pointing triangle) 70/30 and (black lower right triangle) 80/20 at irradiation dose 300 kGy

The effect of the St/AN weight ratio of UFPNR-g-(PS-co-PAN) with monomers content 15 phr at irradiation dose 300 kGy on the 5% weight loss (*T*_d5_) was studied. TGA thermograms are shown in Fig. 18 and the numerical data in Table 4. The results found that an increase in *T*_d5_ of the UFPNR-g-(PS-co-PAN) with raising the St in St/AN weight ratio of 20/80, 30/70 and 50/50 cause an increase in *T*_d5_ from 351 to 354 and to 356 °C, respectively, due to the increase of monomer conversion and grafting efficiency. The sufficient irradiation dose of 300 kGy can activate the double bonds of NR structure and coagent to generate highly reactive radicals and form a three-dimensional network. This leads to higher heat energy consumption to destructive the polymer chains. However, raising the amount of St in St/AN weight ratio up to 70/30 and 80/20 resulted in *T*_d5_ decline to 352 and 349 oC, respectively. As the percentage of St is increased (≥ 70 wt%) the aromatic benzene rings of the St units provide lower irradiation resistance.

**Table 4:** Degradation temperature at 5% weight loss (*T*_d5_) at various monomer content

Sample	Monomer contents (phr)	ST/AN (wt%)	Irradiation doses (kGy)	*T*_d5_ (°C)
Unmodified NR	–	–	300	334
UFPNR-g-(PS-co-PAN)	15	20/80	300	351
UFPNR-g-(PS-co-PAN)	15	30/70	300	354
UFPNR-g-(PS-co-PAN)	15	50/50	300	356
UFPNR-g-(PS-co-PAN)	15	70/30	300	352
UFPNR-g-(PS-co-PAN)	15	80/20	300	349

The physical properties of PS remain relatively stable even after high doses of irradiation, this phenomenon can generate low reactivity free radicals by the resonance in aromatic rings through to the backbone chains, which could induce chain scission in a NR molecule to counterbalance the effects of crosslinking (Bee et al. 2018; Burlant et al. 1962). Moreover, the benzene rings also cause high stiffness and low flexibility of NR chain resulting in block radical neighbour chains or partner macroradical to form crosslink network leading to chain scission of rubber chain (Bandzierz et al. 2018). Therefore, thermal stability can imply this phenomenon predominantly undergo chain scission in the NR backbone led to give the shorter chains and reduce the degree of crosslinked network when subjected to high energy radiation.

#### The glass transition temperature of UFPNR-g-(PS-co-PAN)

**Fig. 19:**
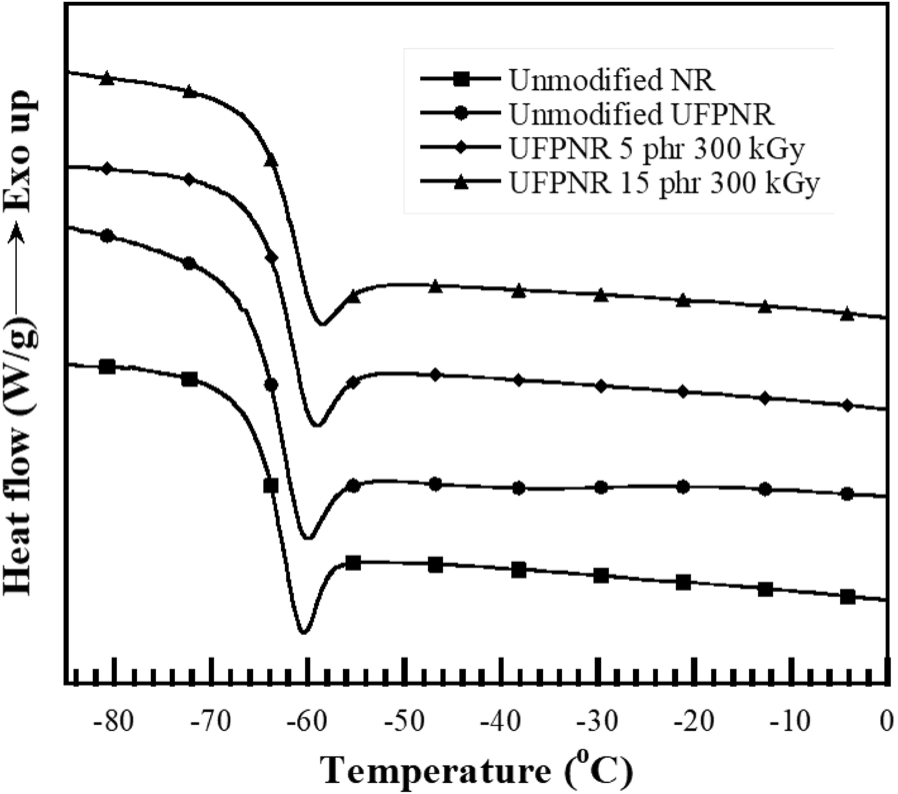
Glass transition temperature of (black square) unmodified NR, (black circle) unmodified UFPNR at irradiation dose 300 kGy, and UFPNR-g-(PS-co-PAN) at monomers content (black diamond) 5 phr and (black up-pointing triangle) 15 phr at irradiation dose 300 kGy

*T*_g_ was determined by a DSC as shown in Fig. 19 and the numerical data are tabulated in Table 5. The results found that the *T*_g_ of virgin NR is − 64 °C and increased to − 63 °C when irradiated with electron beam 300 kGy. In the high irradiation a denser three-dimensional network structure in radiated UFPNR-g-(PS-co-PAN) is produced with restriction of the chain movement and lesser number of free chains available for glassy to rubbery transition (Rezaei Abadchi and Jalali-Arani 2014; Taewattana et al. 2018). Therefore, modified UFPNR with monomers content of 5 or 15 phr at 50/50 of St/AN weight ratio radiated at a dose of 300 kGy are representative to investigate the *T*_g_ which are − 62 °C. It is possible that the bulky styrenic benzene ring of PS side chains can interact with neighbor chains and restrict their rotational freedom. In addition, intermolecular forces in grafted copolymers increased due to the introduction of polar nitrile groups in PAN at interface of NR and may cause movement limitation of NR chain. However, the effect of irradiation dose on *T*_g_ of modified UFPNRs was slightly increased compared with un-irradiated NR, which is about − 64 °C, agreeing with previous work (Lin et al. 2021; Taewattana et al. 2018; Wongkumchai et al. 2021).

**Table 5:** Glass transition temperature (*T*_g_) of UFPNR

Sample	Monomer contents (phr)	ST/AN ratio (wt%)	Irradiation dose (kGy)	*T*_g_ (°C)
Unmodified NR	–	–	–	− 64
Unmodified UFPNR	–	–	300	− 63
UFPNR-g-(PS-co-PAN)	5	50/50	300	− 62
UFPNR-g-(PS-co-PAN)	15	50/50	300	− 62

## Conclusions

The modified NR latex, DPNR-g-(PS-co-PAN), was successfully prepared by grafting St-AN co-monomers onto DPNR latex via emulsion copolymerization confirmed by FTIR spectra and ^1^H NMR spectra. The addition of St monomer up to St/AN weight ratio of 80/20 at monomer content of 15 phr provided the highest monomer conversion and grafting efficiency at 89 and 86%, respectively. Moreover, the DPNR-g-(PS-co-PAN) having desirable core–shell morphology is confirmed by TEM micrographs. The obtained modified NR has qualified to produce modified UFPNR by radiation with an electron beam in the presence of DTMPTA as a coagent followed by the spray drying process to produce UFPNR. The results revealed that the irradiation dose 300 kGy resulted in improved solvent resistance of the UFPNR-g-(PS-co-PAN) by reducing swelling ratio and molecular weight between crosslinks. The grafting and irradiation can improve the morphology of UFPNR particles, which are relatively spherical and show a non-aggregated smooth surface with a particle size of approximately 4.4 ± 1.8 µm. Thermal stability, i.e., degradation temperature at 5% weight (*T*_d5_) of the UFPNR modified by grafted with various St/AN weight ratios at monomer content, 15 phr was found to be in a range of 349 to 356 °C. On the contrary, the *T*_g_ of modified UFPNR and unmodified UFPNR were not significantly enhanced and consequently they maintain the elastomeric properties of the UFPNR. Furthermore, the contact angle measurement result revealed that the modified UFPNR is suitable for utilizing as toughening filler for wide polarity and types of polymers.

## Data Availability

All data analyzed during this study are included in this article.
